# Cryptic Genomic Rearrangements in Three Patients with 46,XY Disorders of Sex Development

**DOI:** 10.1371/journal.pone.0068194

**Published:** 2013-07-08

**Authors:** Maki Igarashi, Vu Chi Dung, Erina Suzuki, Shinobu Ida, Mariko Nakacho, Kazuhiko Nakabayashi, Kentaro Mizuno, Yutaro Hayashi, Kenjiro Kohri, Yoshiyuki Kojima, Tsutomu Ogata, Maki Fukami

**Affiliations:** 1 Department of Molecular Endocrinology, National Research Institute for Child Health and Development, Tokyo, Japan; 2 Department of Endocrinology, Metabolism and Genetics, The Vietnam National Hospital of Pediatrics, Hanoi, Vietnam; 3 Department of Gastroenterology and Endocrinology, Osaka Medical Center and Research Institute for Maternal and Child Health, Osaka, Japan; 4 Department of of Maternal-Fetal Biology, National Research Institute for Child Health and Development, Tokyo, Japan; 5 Department of Nephro-Urology, Nagoya City University Graduate School of Medical Sciences, Nagoya, Japan; 6 Department of Urology, Fukushima Medical University School of Medicine, Fukushima, Japan; 7 Department of Pediatrics, Hamamatsu University School of Medicine, Hamamatsu, Japan; Institut Jacques Monod, France

## Abstract

**Background:**

46,XY disorders of sex development (46,XY DSD) are genetically heterogeneous conditions. Recently, a few submicroscopic genomic rearrangements have been reported as novel genetic causes of 46,XY DSD.

**Methodology/Principal Findings:**

To clarify the role of cryptic rearrangements in the development of 46,XY DSD, we performed array-based comparative genomic hybridization analysis for 24 genetic males with genital abnormalities. Heterozygous submicroscopic deletions were identified in three cases (cases 1–3). A ∼8.5 Mb terminal deletion at 9p24.1–24.3 was detected in case 1 that presented with complete female-type external genitalia and mental retardation; a ∼2.0 Mb interstitial deletion at 20p13 was identified in case 2 with ambiguous external genitalia and short stature; and a ∼18.0 Mb interstitial deletion at 2q31.1–32 was found in case 3 with ambiguous external genitalia, mental retardation and multiple anomalies. The genital abnormalities of case 1 could be ascribed to gonadal dysgenesis caused by haploinsufficiency of *DMRT1*, while those of case 3 were possibly associated with perturbed organogenesis due to a deletion of the *HOXD* cluster. The deletion in case 2 affected 36 genes, none of which have been previously implicated in sex development.

**Conclusions/Significance:**

The results indicate that cryptic genomic rearrangements constitute an important part of the molecular bases of 46,XY DSD and that submicroscopic deletions can lead to various types of 46,XY DSD that occur as components of contiguous gene deletion syndromes. Most importantly, our data provide a novel candidate locus for 46,XY DSD at 20p13.

## Introduction

46,XY disorders of sex development (46,XY DSD) are genetically heterogeneous conditions that result from the impaired production or function of androgens, or from defective organogenesis of external genitalia [1]. To date, several genes such as *SRY*, *AR*, *SRD5A2,* and *SOX9* have been identified as causative genes for 46,XY DSD, although mutations in these genes account for only a minor fraction of the molecular causes of these conditions [1], [2].

Recent advances in microarray technology, including comparative genomic hybridization (CGH) analysis and single nucleotide polymorphism (SNP) genotyping, have enabled researchers to identify genomic rearrangements in individuals with apparently normal karyotypes [3]. Cryptic genomic rearrangements can lead to developmental disorders, although they can also occur as benign polymorphisms [4]. To date, CGH analysis and SNP genotyping have been carried out for patients with 46,XY DSD, identifying multiple submicroscopic deletions and duplications [5], [6], [7]. Such rearrangements frequently affected coding exons or regulatory regions of known DSD-associated genes including *SF1*, *SOX9* and *DMRT1,* or exons of candidate genes including *KANK1* and *ZEB2* [5], [6], [7]. These data suggest that genomic abnormalities at various chromosomal loci may underlie 46,XY DSD.

To clarify the role of cryptic genomic rearrangements in the development of 46,XY DSD, we performed copy-number analyses for 24 patients. The results provide novel insights into the molecular basis of 46,XY DSD.

## Subjects and Methods

### Ethics Statement

This study was approved by the Institutional Review Board Committee at the National Center for Child Health and Development. After obtaining written informed consent from the parents, peripheral blood samples were collected from the patients. When possible, blood samples were also obtained from the parents.

### Patients

The study population comprised 24 patients with 46, XY DSD, including nine cases with complete female-type external genitalia, five with ambiguous genitalia and 10 with male-type external genitalia with hypospadias (Table 1). None of the 24 patients had a family history of DSD or a history of prenatal exposure to specific environmental pollutants. G-banding analysis showed a normal 46,XY karyotype in all patients. Mutations in the coding regions of known DSD-causative genes, *SRY, AR, SRD5A2, SF1, WNT4, SOX9, WT1, BNC2, DMRT1, HSD17B3,* and *MAP3K1,* were excluded by sequence analyses.

**Table 1:** Patients analyzed in the present study.

Cases	Karyotype	Ethnic origin	External genitalia	Additional clinical features
1	46,XY	Japanese	Female	Mental retardation, schizophrenia
2	46,XY	Vietnamese	Ambiguous	Short stature
3	46,XY	Vietnamese	Ambiguous	Short stature, mental retardation, multiple anomalies
4	46,XY	Japanese	Female	
5	46,XY	Japanese	Female	Upper limb anomalies
6	46,XY	Japanese	Female	
7	46,XY	Japanese	Female	Short stature
8	46,XY	Japanese	Female	
9	46,XY	Japanese	Female	
10	46,XY	Japanese	Female	
11	46,XY	Japanese	Female	Agenesis of the corpus callosum, short palpebral fissures
12	46,XY	Japanese	Ambiguous	
13	46,XY	Japanese	Ambiguous	
14	46,XY	Indian	Ambiguous	
15	46,XY	Japanese	Male with HS	
16	46,XY	Japanese	Male with HS	
17	46,XY	Japanese	Male with HS	
18	46,XY	Japanese	Male with HS	
19	46,XY	Japanese	Male with HS	
20	46,XY	Japanese	Male with HS	
21	46,XY	Japanese	Male with HS	
22	46,XY	Japanese	Male with HS	
23	46,XY	Japanese	Male with HS	
24	46,XY	Vietnamese	Male with HS	

DSD, disorders of sex development; HS, hypospadias.

### CGH Analysis

Genomic DNA samples were subjected to CGH analyses using a catalog human array (4×180 k format, Agilent Technologies, Palo Alto, CA), according the manufacturer’s instructions. The sizes and positions of the genomic rearrangements were analyzed using the UCSC genome browser (http://genome.ucsc.edu/; February 2009, hg19, build 37). In the present study, we focused on copy-number alterations with a physical size of more than 1.5 Mb, which have a higher probability of being associated with disease phenotypes [8]. Deletions and duplications registered in the database of genomic variants (http://projects.tcag.ca/variation/) were excluded as benign polymorphisms.

## Results

### CGH Analysis

**Figure 1:**
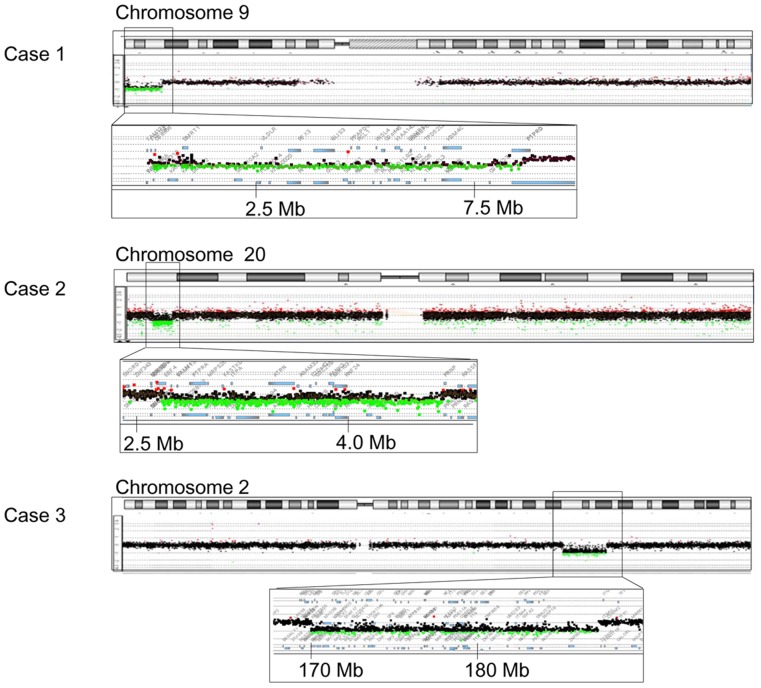
Cryptic heterozygous deletions in cases 1–3. CGH analysis identified heterozygous deletions in cases 1–3. The black, red, and green dots denote signals indicative of the normal, the increased (> +0.5) and the decreased (< − 1.0) copy-numbers, respectively. Genomic positions correspond to the human genome reference assembly (UCSC Genome Browser, February 2009, hg19, build 37). The names of the genes affected by the deletions are shown in Table 2.

We identified heterozygous submicroscopic deletions in three cases (cases 1–3; Fig. 1). The deletions affected several genes (Table 2). Case 1 harbored a ∼8.5 Mb terminal deletion at 9p24.1–24.3 that encompassed *DMRT1*, in addition to 39 other genes. Case 2 carried a ∼2.0 Mb interstitial deletion at 20p13 that included 36 genes. Case 3 had a ∼18.0 Mb interstitial deletion at 2q31.1–32.1 that affected the entire *HOXD* cluster (*HOXD 1–13*), and 84 other genes. The parents of case 2 did not carry the deletion, whereas the parental samples of cases 1 and 3 were not available for genetic analyses.

**Table 2:** Genes affected by the cryptic deletions.

Case 1	Case 2	Case 3	
C9orf66	EBF4	BBS5	CHN1
DOCK8	CPXM1	KBTBD10	ATF2
KANK1	C20orf141	FASTKD1	ATP5G3
DMRT1	FAM113A	PPIG	KIAA1715
DMRT3	TMEM239	CCDC173	EVX2
DMRT2	VPS16	SSB	HOXD1-13
SMARCA2	PTPRA	C2orf77	MTX2
FLJ35024	GNRH2	PHOSPHO2	LOC375295
VLDLR	MRPS26	KLHL23	HNRNPA3
KCNV2	OXT	METTL5	LOC100506866
KIAA0020	AVP	UBR3	NFE2L2
RFX3	LOC100134015	MYO3B	NR_026966
GLIS3	UBOX5	LOC440925	AGPS
C9orf68	FASTKD5	LOC285141	TTC30B
SLC1A1	SLC4A11	SP5	TTC30A
SPATA6L	C20orf194	NR_046248	PDE11A
AK3	DDRGK1	GAD1	SNORD77
CDC37L1	ITPA	GORASP2	OSBPL6
RCL1	SLC4A11	TLK1	DFNB59
C9orf46	C20orf194	METTL8	FKBP7
JAK2	ATRN	DCAF17	PLEKHA3
INSL6	GFRA4	CYBRD1	LOC100506866
INSL4	ADAM33	DYNC1I2	TTN
RLN2	SIGLEC1	SLC25A12	CCDC141
RLN1	HSPA12B	HAT1	SESTD1
C9orf46	C20orf27	METAP1D	ZNF385B
CD274	CDC25B	DLX1	CWC22
PDCD1LG2	C20orf29	DLX2	UBE2E3
KIAA1432	SPEF1	ITGA6	ITGA4
ERMP1	CENPB	PDK1	CERKL
MLANA	MAVS	RAPGEF4-AS1	NEUROD1
KIAA2026	PANK2	RAPGEF4	SSFA2
RANBP6	RNF24	ZAK	PPP1R1C
IL33	SMOX	MLK7-AS1	PDE1A
TPD52L3	LOC728228	CDCA7	DNAJC10
UHRF2	ADRA1D	SP3	FRZB
GLDC		OLA1	NCKAP1
KDM4C		LOC285084	PDE1A
C9orf123		CIR1	DUSP19
PTPRD		SCRN3	NUP35
		GPR155	ZNF804A
		WIPF1	FSIP2
		CHRNA1	

### Clinical Features of Deletion-positive Patients

Case 1 was a genetic male born to non-consanguineous Japanese parents. This patient had complete female-type external genitalia and was raised as a female. This patient exhibited mental retardation and behavioral problems and was diagnosed as having schizophrenia. At 17 years of age, this patient was referred to our clinic because of primary amenorrhea. Clinical analysis detected no dysmorphic facial features or cardiac/renal abnormalities. Abdominal ultrasonography delineated a uterus. Blood endocrine tests indicated primary hypogonadism (Table 3). At 17 years of age, the patient underwent gonadectomy. Histological analyses showed bilateral streak gonads with ovarian ducts. The parents were clinically normal.

**Table 3:** Clinical and laboratory findings of cases 1–3.

Cases		Case 1	Case 2	Case 3
Molecular analyses				
Karyotype (G-banding)		46,XY	46,XY	46,XY
Genomic rearrangement		Deletion	Deletion	Deletion
Genomic position of the deletion		9p24.1–24.3	20p13	2q31–32
Size of the deletion		∼8.5 Mb	∼2.0 Mb	∼18.0 Mb
Parental origin of the deletion		Unknown	*de novo*	Unknown
Clinical features				
External genitalia		Female-type genitalia	Ambiguous	Ambiguous
Mental retardation		Yes	No	Yes
Growth failure/Short stature		No	Yes	Yes
Dysmorphic facial appearance		No	No	Yes
Additional features		Schizophrenia	Delayed bone age	Skeletal anomalies
				Brain anomalies
				Convulsion
Endocrine data^a^				
Age at examination		17 y	4.5 y	at birth
LH (mIU/mL)		*17.4* (0.2–2.2)	**0.01** (0.2–1.9)	
FSH (mIU/mL)		*101.1* (0.6–4.8)		
Testosterone (nmol/L)		**0.71** (9–32)	**0.01** (0.2–0.5)	4.9 (<12)
GH after physical exercise (ng/mL)			**1.5** (3.0–28.3)	

DSD, disorders of sex development; MP, micropenis; HS, hypospadias; CO, cryptorchidism.

The hormone values below the reference range are boldfaced, and those above the reference range are italicized.

a Reference values of the age-matched control individuals are shown in the parenthesis.

**Figure 2:**
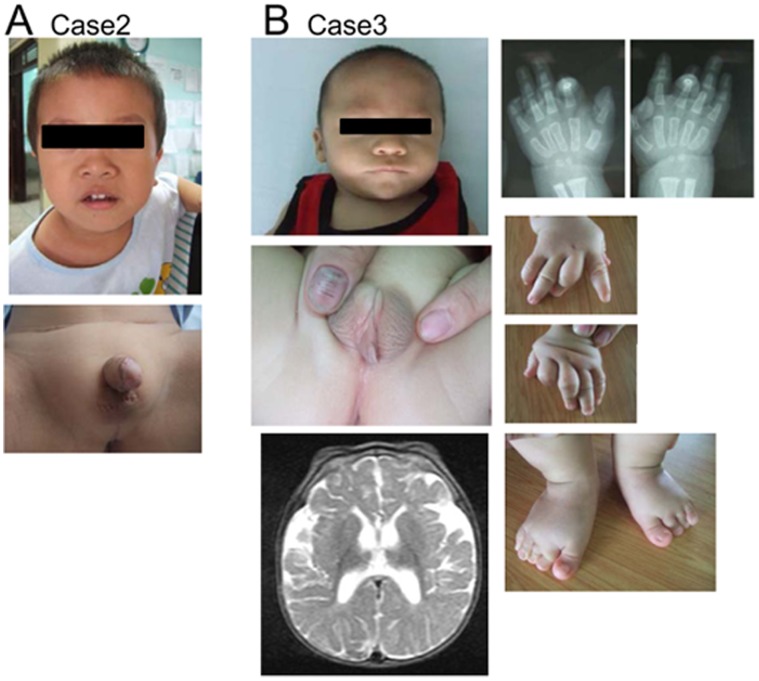
Clinical features of cases 2 and 3. A. Clinical findings of case 2 at 4.5 years of age. Images of the craniofacial region and external genitalia (after surgical intervention) are shown. B. Clinical findings of case 3 at 11 months of age. Multiple facial dysmorphisms and limb anomalies including syndactyly and camptodactyly are shown. Brain magnetic resonance imaging indicates delayed myelination, hypogenesis of the corpus callosum and prominent ventricular and CSF spaces. The parents of cases 2 and 3 have given written informed consent, as outlined in the PLOS consent form, to publication of the photographs of the patients.

Case 2 was a genetic male born to non-consanguineous Vietnamese parents. At birth, this patient exhibited a micropenis, cryptorchidism, and distal hypospadias. Abdominal ultrasonography detected bilateral testes (12×6 mm) in inguinal canals. The uterus and ovaries were absent. This patient was raised as a boy and underwent surgical intervention for hypospadias and cryptorchidism at 4 years and 2 months and at 4 years and 3 months of age, respectively. On his visit at 4.5 years of age, the patient showed a penis with a stretched length of 3 cm, and left testis (12×9 mm) in the scrotum and right testis (13×6 mm) in the inguinal canal (Fig. 2). He had no dysmorphic facial features (Fig. 2). He showed short stature (89 cm, −2.9 SD) and delayed bone age (equivalent to 2 years of age). His mental development was normal. Blood endocrine tests at 4.5 years of age showed low levels of luteinizing hormone and testosterone (Table 3). His growth hormone levels were within the normal range at the baseline, but remained low after physical exercise. His parents were clinically normal and had normal statures.

Case 3 was born to non-consanguineous Vietnamese parents at 40 weeks of gestation with a birth weight of 2.0 kg (−3.7 SD). At birth, this patient manifested severe micropenis and hypospadias (Fig. 2). Bilateral testes were palpable in the scrotum, and uterus and ovaries were absent. Thus, this patient was raised as a boy. In addition to genital abnormalities, he exhibited multiple anomalies of the fingers and toes, i.e., camptodactyly and flexion contracture of the proximal interphalangeal joint of the right index and left ring fingers, cutaneous syndactyly of the 2nd and 3rd toes and medial deviation of the 4th toe in the right foot, lateral deviation of the 2nd toe and medial deviation the 4th toe in the left foot, and overriding of the 4th toe on the third toe in both feet (Fig. 2). Furthermore, he showed dysmorphic facial features such as ptosis and micrognathia (Fig. 2). His blood testosterone level at birth was within the normal range (Table 3). On examination at 11 months of age, he showed obvious growth retardation (body weight; 6.0 kg, <−3.0 SD) and developmental delay (DQ <30). At one year of age, he presented with an episode of febrile convulsion. Brain magnetic resonance imaging detected delayed myelination, hypogenesis of the corpus callosum, and prominent ventricular and CSF spaces (Fig. 2). His parents were clinically normal.

## Discussion

We identified cryptic heterozygous deletions with physical sizes of more than 1.5 Mb in three of the 24 patients with 46,XY DSD. The results support the notion that submicroscopic genomic rearrangements constitute a portion of causative mechanisms for 46,XY DSD [5], [6], [7]. Furthermore, molecular and clinical data of the three cases imply that cryptic deletions can cause DSD as components of contiguous gene deletion syndromes. Since array-based CGH analysis and SNP genotyping can detect copy-number alterations across the entire genome in a single assay, these methods should be considered for patients with 46,XY DSD, particularly for those with additional clinical manifestations.

**Figure 3:**
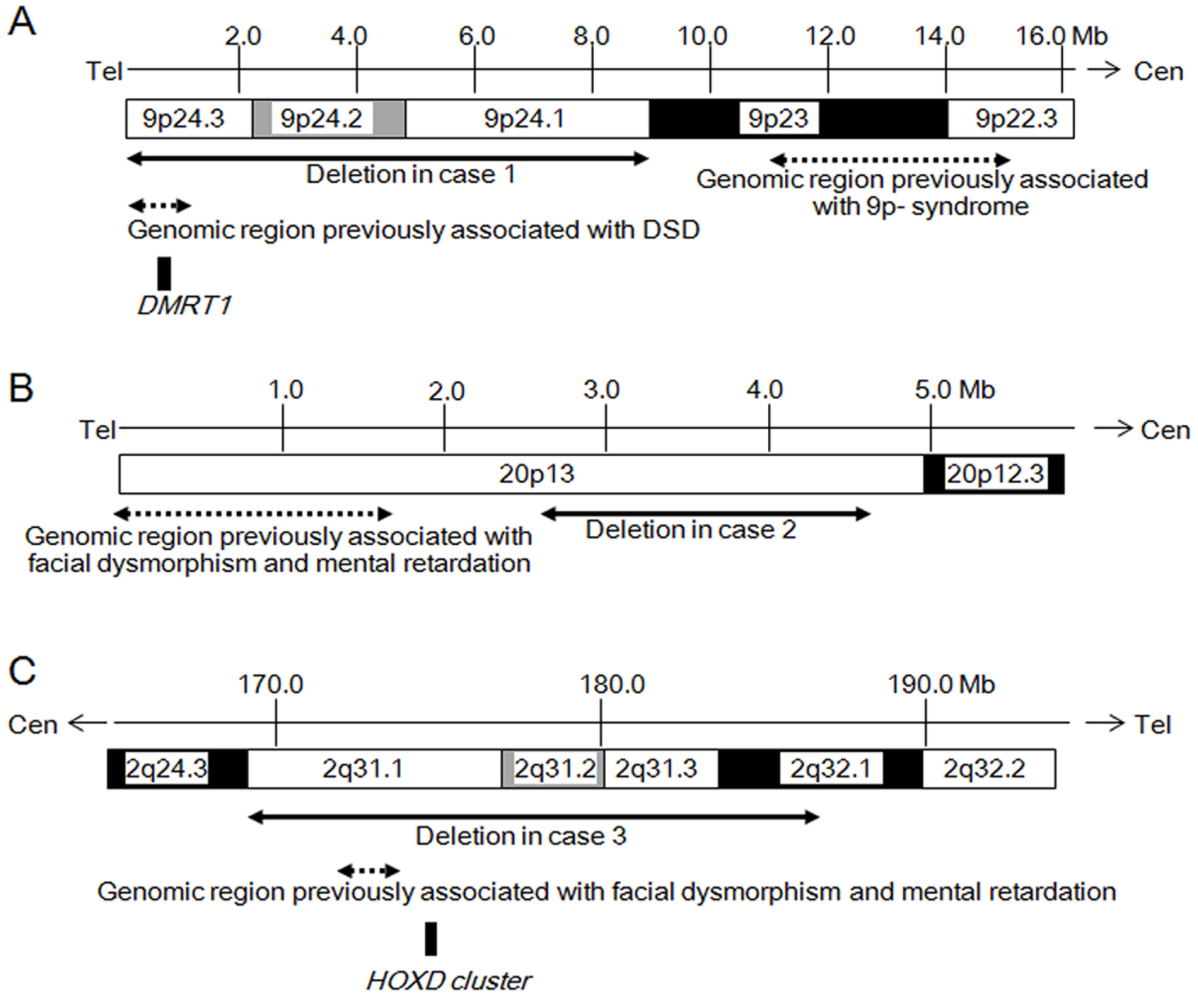
Schematic representation of the genomic regions around the deletions. A. Terminal part of the short arm of chromosome 9. The black arrow denotes the deletion identified in case 1. The dotted arrows indicate the genomic intervals associated with DSD and for 9p- syndrome [13]. The black box indicates the position of *DMRT1* that is likely to be associated with DSD in case 1. B. Terminal part of the short arm of chromosome 20. The black arrow denotes the deletion in case 2. The dotted arrow indicates the genomic region associated with facial dysmorphism and mental retardation [20]. C. The 2q24.3–2q32.2 region. The black arrow denotes the deletion in case 3. The dotted arrow indicates the genomic region associated with facial dysmorphism and mental retardation [22]. The black box indicates the position of the *HOXD* cluster possibly associated with DSD in case 3.

Case 1 had a ∼8.5 Mb heterozygous deletion at 9p involving 40 genes. Of the 40 genes, *DMRT1* is known to encode a male specific transcriptional regulator with a conserved zinc finger-like DNA-binding domain [9], [10]. Since mouse *Dmrt1* has been implicated in testicular differentiation [11], and intragenic deletions of human *DMRT1* have been identified in 46, XY patients with gonadal dysgenesis [6], [12], it appears that DSD in case 1 results from haploinsufficiency of *DMRT1*. These data argue for the assumption that heterozygous deletions involving the coding exons and/or the upstream region of *DMRT1* account for a substantial part of the etiology of complete and partial gonadal dysgenesis in individuals with 46, XY karyotype [6], [12], [13], [14]. Furthermore, our results provide additional information about other disease-associated loci. First, deletions at 9p22.3–23 are known to cause various malformations, such as craniofacial abnormalities, cardiac defects, and dysplastic kidneys, which are collectively referred to as the 9p- syndrome [13]. Lack of clinical manifestations of the 9p- syndrome in case 1 implies that the gene(s) responsible for this syndrome is not located in the ∼8.5 Mb terminal region. This is consistent with previous studies which mapped the critical region of this syndrome to a genomic interval approximately 11–15 Mb from the telomere (Fig. 3A) [13]. Second, terminal deletions at 9p have previously been associated with mental retardation [13]. Our data suggest that a gene involved in brain development resides in the ∼8.5 Mb terminal region that is deleted in case 1 (Fig. 3A).

Case 2 had a *de novo* ∼2.0 Mb interstitial deletion at 20p13, which has not been identified previously in patients with DSD. Furthermore, none of the 36 genes affected by the deletion have been associated with sex development. These results, in conjunction with previous reports of hypomasculinized external genitalia in a patient with a ≥6 Mb deletion at 20p13-12.3 [15] and in a patient with a 20p11.2-pter deletion [16], indicate that the genomic interval spanning ∼2.7–4.7 Mb from the telomere (deleted in case 2 and in the two aforementioned patients) encompasses a novel causative gene for DSD (Fig. 3B). However, the penetrance of DSD in males with 20p13 deletions appears to be low, because genital abnormalities have been described only in a small percentage of patients with such deletions [16], [17], [18], [19]. It might also be possible that the 20p13 and/or the 2q31.1–32 deletion has unmasked a recessive mutation of the testis development gene(s) on the structurally normal homologous chromosome, leading to DSD. In addition, the deletion of case 2 seems to harbor a gene that is indispensable for growth, because short stature was observed in case 2, as well as in most patients with partial monosomy of 20p [16], [17], [18], [19]. In this regard, although case 2 showed impaired growth hormone secretion after exercise, it remains to be clarified whether short stature in patients with 20p deletions is ascribed to growth hormone deficiency. Furthermore, unlike patients with terminal deletions of 20p [16], [17], [18], [19] case 2 showed no facial dysmorphism or mental retardation. These data indicate that a gene(s) involved in the development of the craniofacial region and brain resides within the 0–2.7 Mb interval from the telomere that is preserved in case 2. Consistent with this, facial abnormalities and developmental delay have been reported in two patients harboring 20p terminal deletions of less than 1.7 Mb [20] (Fig. 3B). Importantly, the deletion in case 2 includes *OXT* and *AVP* that are predicted to play a role in social behavior [21]. Lack of social dysfunction in case 2 indicates that haploinsufficiency of *OXT* and *AVP* permits normal psychosocial development at least in childhood. However, this notion awaits further investigation.

Case 3 had a ∼18.0 Mb interstitial deletion at 2q31.1–32.1. Clinical manifestations of case 3 including finger/toe anomalies, mental retardation and facial dysmorphism are compatible with the 2q31 microdeletion syndrome, a well-established contiguous gene deletion syndrome [22]. Notably, abnormal formation of the external genitalia has been reported in both male and female patients carrying 2q31 deletions [23], [24]. Previous studies have attributed the skeletal anomalies of 2q31 microdeletion syndrome to haploinsufficiency of the *HOXD* cluster [22], [25], and mental retardation and craniofacial abnormalities to deletions of certain genes located within the genomic interval spanning 174–175 Mb from the 2q telomere [22] (Fig. 3C). In this regard, while skeletal abnormalities are obviously milder in case 3 than the previously reported patients with deletions involving *HOXD* genes [25], this would be consistent with the assumption that haploinsufficiency of developmental genes is frequently associated with a broad phenotypic spectrum [26]. Since mouse *Hoxd* genes have been shown to play a role in the formation of external genitalia by regulating multiple target genes, genital abnormalities of 2q31 microdeletion syndrome could be associated with haploinsufficiency of *HOXD* genes [25], [27]. Indeed, the phenotype of case 3, such as hypomasculinized external genitalia without cryptorchidism and a normal blood testosterone value at birth, is indicative of perturbed organogenesis of the external genitalia rather than impaired hormone production in the gonads. However, since DSD has been described for only a small subset of males with 2q31 deletions [22], [23], [24], [25], impaired sex development in case 3 may be caused by other unknown genetic or environmental factors.

In summary, we identified cryptic genomic rearrangements in three of 24 individuals with 46,XY DSD. It appears that the genital abnormalities of case 1 result from gonadal dysgenesis due to haploinsufficiency of *DMRT1*, while those of case 3 can be ascribed to perturbed organogenesis due to the deletion of the *HOXD* cluster. These data suggest that submicroscopic deletions can lead to various types of 46,XY DSD that occur as components of contiguous gene deletion syndromes. Moreover, the results obtained from case 2 provide a novel candidate locus for 46,XY DSD at 20p13. Further copy-number analyses on patients with 46,XY DSD and functional assays for genes involved in the genomic rearrangements will help to clarify novel causative mechanisms for 46,XY DSD.
